# Supplementary material for: The Microbiology and Geochemistry of the Shallow‐Water Hydrothermal Vents of the Gulf of Naples, Italy
Source: Environ Microbiol. 2026 Apr 14;28(4):e70296. doi: 10.1111/1462-2920.70296 (PMC13078919; doi:10.1111/1462-2920.70296)
Supplement: Supplementary file 1 — Figure S1: Correlation plot between all the variables measured in the present study Figure S2: Beta‐diversity analysis based on Non‐metric multidimensional scaling (nMDS) using the Unweighted Jaccard similarity index coloured by (1) Region and (2) Geochemistry of the fluids Table S1: Table of the major ASVs (above 2%) at each of the sampling sites (Abundance % is reported as an average between fluid and sediment samples. Taxonomic classifications of Silva, Ez taxon, and NCBI nucleotide database. Table S2: Envfit results against nMDS1, nMDS2‐ Jaccard weighted. One star (*); p value less than 0.01, two stars (**) p value is less than 0.001. Only variables with a p value of less than 0.05 are marked in bold and considered statistically significant. Table S3: Envfit results against nMDS1, nMDS2‐ Jaccard unweighted. One star (*); p value less than 0.05, two stars (**) p value is less than 0.01. Only variables with a p value of less than 0.05 are marked in bold and considered statistically significant. [file EMI-28-e70296-s001.docx]

The microbiology and geochemistry of the shallow-water hydrothermal vents of the Gulf of Naples, Italy

Bernardo Barosa^1†^, Carmela Celentano^1^, Flavia Migliaccio^1^, Sara Claudia Diana^1,2^, Ana Clara Pelliciari Silva^1^, Matteo Selci^1,3^, Luca Tonietti^1,4,5^, Deborah Bastoni^1^, Martina Cascone^1^, Alessia Bastianoni^1^, Monica Correggia^1^, Luciano di Iorio^1^, Roy Price^6^, Stefano Caliro^7^, Marco Milazzo^1,2^, Alessandro Aiuppa^1,2^, Costantino Vetriani^3,8^, Angelina Cordone^1^, Donato Giovannelli^1,3,9,10,11*^

1 Department of Biology, University of Naples “Federico II”, Naples, Italy

2 Department of Earth and Sea Sciences, University of Palermo, Italy National Biodiversity Future Center, Palermo, Italy

3 Department of Marine and Coastal Science, Rutgers University, New Brunswick, NJ, USA

4 Department of Science and Technology, University of Naples, Parthenope, Naples, Italy

5 INAF-OAC, Osservatorio Astronomico di Capodimonte, Naples, Italy

6 School of Marine and Atmospheric Sciences, Stony Brook, NY, United States

7 Istituto Nazionale di Geofisica e Vulcanologia(INGV), Napoli, Italy

8 Department of Biochemistry and Microbiology, Rutgers University, New Brunswick, NJ, USA

9 Istituto per le Risorse Biologiche e Biotecnologiche Marine, Consiglio Nazionale delle Ricerche, CNR-IRBIM, Ancona, Italy

10 Earth-Life Science Institute, Tokyo Institute of Technology, Ookayama, Tokyo, Japan

11 Marine Chemistry & Geochemistry Department - Woods Hole Oceanographic Institution, MA, USA

***Correspondence:** Donato Giovannelli [donato.giovannelli@unina.it](mailto:donato.giovannelli@unina.it)

**Supplementary figure 1-** Correlation plot between all the variables measured in the present study


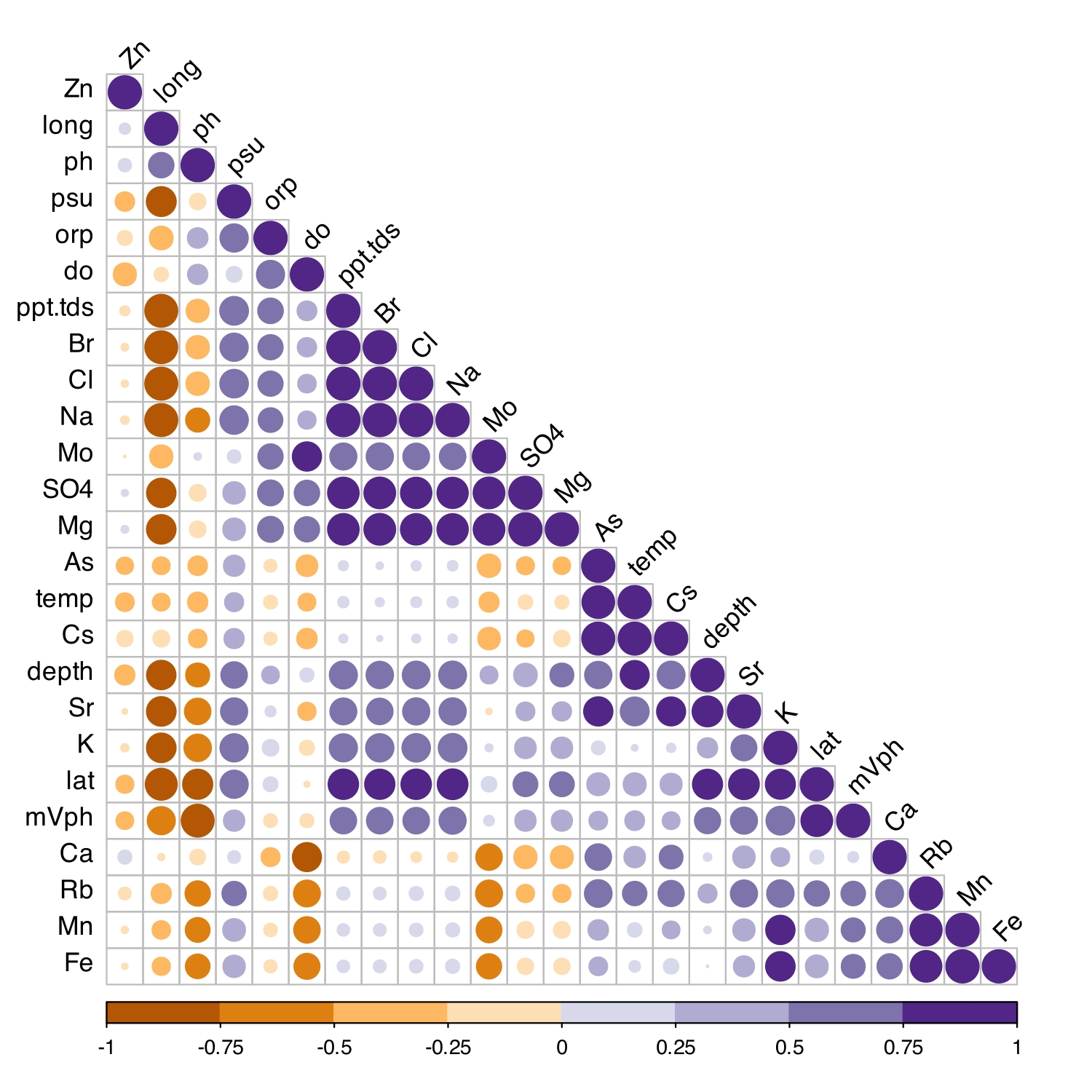


**Supplementary figure 2-** Beta-diversity analysis based on Non-metric multidimensional scaling (nMDS) using the Unweighted Jaccard similarity index colored by 1) Region and 2) Geochemistry of the fluids


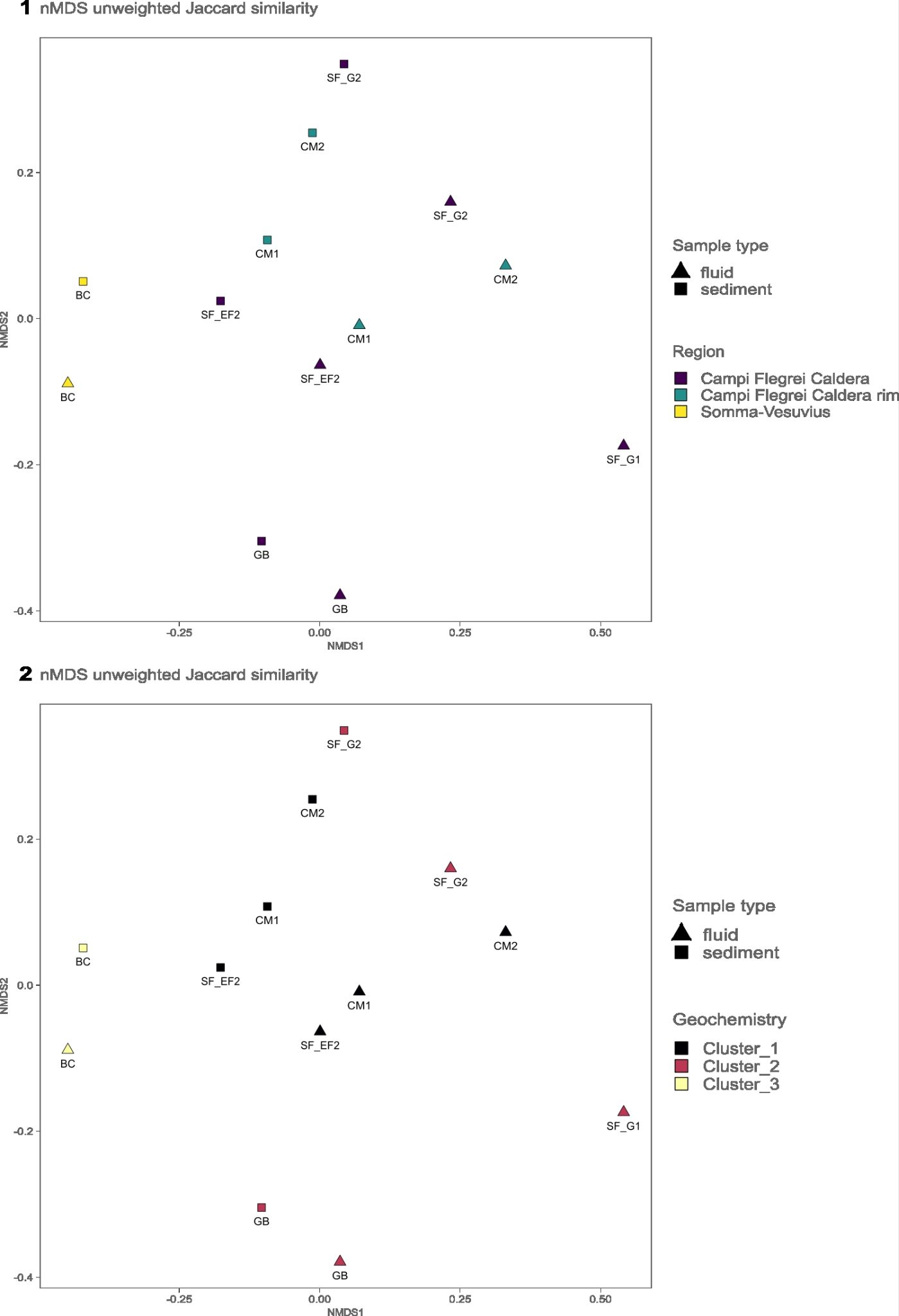


**Supplementary table 1**. Table of the major ASVs (above 2 %) at each of the sampling sites (Abundance % is reported as an average between fluid and sediment samples. Taxonomic classifications of Silva, Ez taxon, and NCBI nucleotide database.

| **ASV** | **Code** | **Abundance**  **(%)** | **Silva classification** | **Ez taxon classification** | **Similarity (%)** | **Accession** | **NCBI nucleotide**  **classification** | **Similarity**  **(%)** | **Accession** | **Environment** |
| --- | --- | --- | --- | --- | --- | --- | --- | --- | --- | --- |
| TACGGAGGGTGCAAGCGTTATCCGGATTCATTGGGTTTAAAGGGTGCGCAGGCGGACTTTTAAGTCAGTGGTGAAATCCCGGGGCTCAACCCCGGAACTGCCATTGATACTGAAAGTCTTGAGTTTGGTTGAAGTAGGCGGAATGTAGCATGTAGCGGTGAAATGCTTAGATATGCTACAGAACACCGATCGCGAAGGCAGCTTACTAAACCAATACTGACGCTCAGGCACGAAAGCGTGGGGAGCGAACAGGATTAGATACCCTGGTAGTCCACGCCGTAAACTATGATCACTCGTTGTTGGCGATACACAGTCAGCGACCAAGCGAAAGCGATAAGTGATCCACCTGGGGAGTACGATCGCAAGGTTG | BC | 4..58 | Bacteroidetes_VC2.1_Bac22 | AY197392_s | 98.87 | AY197392 | Uncultured Cytophaga sp. | 100.00 | AB238986.1 | deep-sea cold seep sediments |
| TACGGAGGGTGTAAGCGTTATCCGGAATCATTGGGTTTAAAGGGTCTGTAGGCGGATTGCTAAGTCAGGGGTGAAATCCCACAGCTCAACTGTGGCATTGCCTTTGATACTGGTGATCTTGAGATATAGTGAGGTAGATAGAATGTGTAGTGTAGCGGTGAAATGCATAGATATTACACAGAATACCGATTGCGAAGGCAGTCTACTAACTATCATCTGACGCTGATAGACGAAAGCGTGGGGAGCGAACAGGATTAGATACCCTGGTAGTCCACGCCGTAAACGATGGATACTAGCTGTTGGACTTTAGGGTTCAGTGGCCAAGCGAAAGTGATAAGTATCCCACCTGGGGAGTACGTTCGCAAGAATG | BC | 13.13 | *Flavobacteriaceae* | EU487997_s | 98.65 | EU487997 | Uncultured bacterium clone CK_1C3_19   \|  \| \| --- \| | 98.6 | EU487997.1 | siliciclastic sediment from Thalassia seagrass bed   \|  \| \| --- \| |
| TACGGAGGGTGCAAGCGTTATCCGGAATCATTGGGTTTAAAGGGTGCGTAGGCGGAAATTTAAGTCAGTGGTGAAAGCCCACAGCTCAACTGTGGAACTGCCATTGATACTGAATTTCTTGAATATGATTGAAGTAGGCGGAATGTGTCATGTAGCGGTGAAATGCATAGATATGACACAGAACACCGATAGCGAAGGCAGCTTACTAAGTCATTATTGACGCTGAGGCACGAAAGCGTGGGGAGCGAACAGGATTAGATACCCTGGTAGTCCACGCCGTAAACTATGATTACTCGTTCTTGGCGATACACAGTCAGGGACTAAGCGAAAGTGATAAGTAATCCACCTGGGGAGTACGATCGCAAGGTTG | BC | 9.45 | NS7_marine_group | FQ032827_s   \|  \| \| --- \| | 93.78   \|  \| \| --- \| | FQ032827 | Uncultured marine bacterium clone BIOS04_MAR_15m171   \|  \| \| --- \| | 95.14 | KM223594.1 | Ultra-oligotrophic South Pacific  Ocean |
| TACAGAGGGTGCAAGCGTTATTCGGAATTACTGGGCGTAAAGCGCGCGTAGGCGGATTATTAAGTCAGTTGTGAAAGCCCTGGGCTCAACCTGGGAACTGCATCTGATACTGGTAGTCTAGAGTTTAAGAGAGGGAAGTGGAATTCCAGGTGTAGCAGTGAAATGCGTAGATATCTGGAGGAACATCAGTGGCGAAGGCGACTTCCTGGCTTAAAACTGACGCTGAGGTGCGAAAGCGTGGGTAGCGAACGGGATTAGATACCCCGGTAGTCCACGCCGTAAACGATGTCAACTAGTTGTTGGTCTTATTAAAAAGATTAGTAACGAAGCTAACGCGATAAGTTGACCGCCTGGGGAGTACGGTCGCAAGATTA | BC | 7.83 | *Thiomicrorhabdus* | *Thiomicrorhabdus hydrogeniphila* | 100 | LC010781 | Uncultured bacterium clone D1-STK-23   \|  \| \| --- \| | 98.37 | NR_028679.1   \|  \| \| --- \| | NA   \|  \| \| --- \| |
| TACGTAGGGGGCAAGCGTTGTCCGGATTTACTGGGTGTAAAGGGCGCGTAGGCGGGTTTGTAAGTCAGTGGTGAAATCCTACAGCTTAACTGTAGAACTGCCTTTGATACTGCAGACCTTGAGTACGGAAGAGAGAGGCGGAATTCCAGGTGTAGTGGTGAAATACGTAGATATCTGGAAGAACACCAGAGGCGAAGGCGGTCTCTTGGTCCGTTACTGACGCTGAGGCGCGAAAGCGTGGGGAGCAAACAGGATTAGATACCCTGGTAGTCCACGCCGTAAACGATGAATACTAGGTGTTGGGTTTTTAACTCAGTGCCGCAGCAAACGCATTAAGTATTCCACCTGGGGAGTACGATCGCAAGGTTG | BC | 4.01 | IheB3-7 | JQ579954_s | 97.83 | JQ579954 | *Melioribacter roseus* P3M-2 | 92.49 | NR_074796.1 | surface of a chute, under the flow of hot water coming from an oil exploring well |
| TACGGAGGGGGCTAGCGTTGTTCGGAATTACTGGGCGTAAAGCGCACGTAGGCGGATTATTAAGTGAGGGGTGAAATCCCAGGGCTCAACCCTGGAACTGCCTCTCATACTGGTAGTCTTGAGTTCGAGAGAGGTGAGTGGAATTCCGAGTGTAGAGGTGAAATTCGTAGATATTCGGAGGAACACCAGTGGCGAAGGCGGCTCACTGGCTCGATACTGACGCTGAGGTGCGAAAGCGTGGGGAGCAAACAGGATTAGATACCCTGGTAGTCCACGCCGTAAACGATGAATGCCAGTCGTCGGGCAGTATACTGTTCGGTGACACACCTAACGGATTAAGCATTCCGCCTGGGGAGTACGGTCGCAAGATTA | BC   \|  \| \| --- \| | 3.8 | *Thioclava* | *Thioclava indica* | 98.92 | AUNB01000081 | *Thioclava indica* strain MCCC 1A00513   \|  \| \| --- \| | 98.92 | NR_136454.1 | surface seawater of the Indian Ocean |
| TACGGAGGATCCAAGCGTTATCCGGAATCATTGGGTTTAAAGGGTCCGTAGGTTGATAATTAAGTCAGAGGTGAAATCCTGCCGCTCAACGGTAGAATTGCCTTTGATACTGGTTATCTTGAGTTATTATGAAGTAGTTAGAATATGTAGTGTAGCGGTGAAATGCATAGATATTACATAGAATACCAATTGCGAAGGCAGATTACTAATAATGAACTGACACTGATGGACGAAAGCGTGGGGAGCGAACAGGATTAGATACCCTGGTAGTCCACGCCGTAAACGATGGATACTAGCTGTTCGGATTTCGGTCTGAGTGGCTAAGCGAAAGTGATAAGTATCCCACCTGGGGAGTACGTTCGCAAGAATG | BC | 2.76 | *Arcticiflavibacter* | *Flavivirga amylovorans* | 98.1 | HM475138 | *Wocania arenilitoris* strain HMF6543   \|  \| \| --- \| | 97.84 | NR_159135.1 | Seashore Sand |
| TACAGAGGGTGCAAGCGTTAATCGGAATTACTGGGCGTAAAGCGCGCGTAGGCGGTTAGATAAGTCAGATGTGAAATCCCCGGGCTCAACCTGGGAACTGCACCTGATACTGTCTGGCTAGAGTTTTGGAGAGGGAAGTAGAATTTCAGGTGTAGCGGTGAAATGCATAGAGATCTGAAGGAATACCAGTGGCGAAGGCGACTTCCTGGCCAAAAACTGACGCTGAGGTGCGAAAGCGTGGGTAGCAAACGGGATTAGATACCCCGGTAGTCCACGCCGTAAACGATGTCAACTAGTCGTTGGTTCCCTTGAGGGATCGGTGACGCAGCTAACGCATTAAGTTGACCGCCTGGGGAGTACGGTCGCAAGACTA | BC | 2.73 | *Cocleimonas* | *Cocleimonas flava* | 98.12 | AB495251 | *Cocleimonas flava* strain KMM 3898 | 98.12 | NR_112909.1 | sand snail |
| TACGGAGGGTGCAAGCGTTACTCGGAATCACTGGGCGTAAAGCGCATGCAGGCGGTTTAATAAGTTAGAAGTGAAATCCTACAGCTTAACTGTAGAACTGCTTCTAAAACTGTTAGACTAGAGTCTGGGAGGGGAAGATGGAATTAGTAGTGTAGGGGTAAAATCCGTAGAGATTACTAGGAATACCAAAAGCGAAGGCGATCTTCTGGAACAGTACTGACGCTGAGATGCGAAAGCGTGGGGAGCAAACAGGATTAGATACCCTGGTAGTCCACGCAGTAAACGATGAATGTTAGTCGTCGGGGCACTAGTTGTTTCGGTGATGCAGTTAACACATTAAACATTCCGCCTGGGGAGTACGGTCGCAAGATTA | BC | 2.3 | *Sulfurovum* | Maps-EB01 | 96.51 | AB278150 | *Sulfurovum denitrificans strain eps51* | 92.80 | NR_179236.1 | hydrothermal field |
| TACGGAGGGTGCAAGCGTTGTTCGGAATCACTGGGCGTAAAGGGCGCGCAGGCGGTTTGATTAGTCAGATGTGAAAGCCCACGGCTTAACCGTGGAAGTGCATTTGAAACTGTCAGACTTGAGTATCAGAGGGGAAAGTGGAATTCCCGGTGTAGAGGTGAAATTCGTAGATATCGGGAGGAATACCGGTGGCGAAGGCGACTTTCTGGCTGAATACTGACGCTGAGGCGCGAAAGCGTGGGGAGCAAACAGGATTAGATACCCTGGTAGTCCACGCCGTAAACGATGTCAACTAGGTGTAGGGGGTGTTGATCCCCTCTGTGCCGCAGCTAACGCATTAAGTTGACCGCCTGGGGAGTACGGTCGCAAGATTA | BC | 2.13 | *Desulfobulbus* | AF449227_s | 99.73 | AF449227 | *Desulfobulbus elongatus* strain FP | 97.86 | NR_029305.1 | NA |
| TACGGAGGGTGCAAGCGTTATCCGGAATCATTGGGTTTAAAGGGTCCGTAGGCGGATTGATAAGTCAGAGGTGAAATCCCACAGCTTAACTGTGGCACTGCCTTTGATACTGTTAGTCTTGAATTATATGGAAGTAGATAGAATGTGTAGTGTAGCGGTGAAATGCTTAGAGATTACACAGAATACCGATTGCGAAGGCAGTCTACTACGTATATATTGACGCTAATGGACGAAAGCGTGGGGAGCGAACAGGATTAGATACCCTGGTAGTCCACGCCGTAAACGATGGACACTAGTTGTTGGATTTATTCAGTGACTAAGCGAAAGTGATAAGTGTCCCACCTGGGGAGTACGATCGCAAGATTG | CM1 | 7.98 | *Lutibacter* | *Lutibacter holmesii* | 97.81 | JQ241142 | Uncultured bacterium clone MethaneSIP16-4-44 | 99.73 | GU584454.1 | Marine Hydrocarbon Seeps |
| TACGGAGGGTGTAAGCGTTATCCGGAATCATTGGGTTTAAAGGGTCTGTAGGCGGATTGCTAAGTCAGGGGTGAAATCCCACAGCTCAACTGTGGCATTGCCTTTGATACTGGTGATCTTGAGATATAGTGAGGTAGATAGAATGTGTAGTGTAGCGGTGAAATGCATAGATATTACACAGAATACCGATTGCGAAGGCAGTCTACTAACTATCATCTGACGCTGATAGACGAAAGCGTGGGGAGCGAACAGGATTAGATACCCTGGTAGTCCACGCCGTAAACGATGGATACTAGCTGTTGGACTTTAGGGTTCAGTGGCCAAGCGAAAGTGATAAGTATCCCACCTGGGGAGTACGTTCGCAAGAATG | CM1 | 5.14 | *Flavobacteriaceae* | EU487997_s | 98.65 | EU487997 | Uncultured bacterium clone CK_1C3_19 | 98.65 | EU487997.1 | geochemical habitat on bacterial symbiont |
| TACGGAGGGTGCAAGCGTTATCCGGAATCACTGGGTTTAAAGGGTGCGTAGGTTGTTAGATAAGTCAGAGGTGAAAGCCTGCAGCTAAACTGTAGAACTGCCTTTGATACTGTCTGGCTTGAATTAGGTTGAGGTTGGCGGAATGTGACATGTAGCGGTGAAATGCATAGATATGTCATAGAACACCAAATGCGAAGGCAGCTGACTAGACCTTAATTGACACTGAGGCACGAAAGCGTGGGGAGCGAACAGGATTAGATACCCTGGTAGTCCACGCCCTAAACGATGCTTACTGGATGTGTGCCCTTCGGGGTGCGCATCTGAGGGAAACCATTAAGTAAGCCACCTGGGGAGTACGTCGGCAACGATG | CM1 | 4.21 | *Saprospiraceae* | JQ579834_s | 92.95 | JQ579834 | Uncultured Bacteroidetes bacterium clone FII-TR039 | 92.70 | JQ579834.1 | oil-polluted subtidal sediments |
| TACGGAGGGGGTTAGCGTTGTTCGGAATTACTGGGCGTAAAGCGCACGTAGGCGGACTGGAAAGTTGGGGGTGAAATCCCGGGGCTCAACCCCGGAACTGCCTCCAAAACTTCCAATCTAGAGATCGAGAGAGGTGAGTGGAATTCCAAGTGTAGAGGTGAAATTCGTAGATATTTGGAGGAACACCAGTGGCGAAGGCGGCTCACTGGCTCGATACTGACGCTGAGGTGCGAAAGCGTGGGGAGCAAACAGGATTAGATACCCTGGTAGTCCACGCCGTAAACGATGAGAGCTAGACGTCGGGCAGCATGCTGTTCGGTGTCGCAGTTAACGCATTAAGCTCTCCGCCTGGGGAGTACGGTCGCAAGATTA | CM1 | 3.84 | Rhodobacteraceae | EU491911_s | 99.46 | EU491911 | Uncultured bacterium clone G46 | 100.00 | JX568047.1 |  |
| TACGGAGGATCCAAGCGTTATCCGGAATCATTGGGTTTAAAGGGTCCGTAGGTGGATAATTAAGTCAGAGGTGAAATCCTGCAGCTCAACTGTAGAATTGCCTTTGAAACTGGTTATCTTGAGTTATTATGAAGTAGTTAGAATATGTAGTGTAGCGGTGAAATGCATAGATATTACATAGAATACCAATTGCGAAGGCAGATTACTAATAATATACTGACACTGATGGACGAAAGCGTGGGGAGCGAACAGGATTAGATACCCTGGTAGTCCACGCCGTAAACGATGGTCACTAGCTGTTCGAACTTCGGTTTGAGTGGCTAAGCGAAAGTGATAAGTGACCCACCTGGGGAGTACGTTCGCAAGAATG | CM1 | 3.67 | *Olleya* | *Olleya namhaensis* | 99.73 | Jgi.1108060 | *Olleya* sp. R2A056317 | 99.73 | LR722880.1 | Coastal marine surface water |
| TACGGAGGGTGCAAGCGTTAATCGGAATTACTGGGCGTAAAGGGTACGTAGGCGGTTATTTAAGTCAGATGTGAAATCCCTGGGCTCAACCTAGGAATGGCATCTGATACTGGATAACTTGAGTTTAAGAGAGGAGTGTGGAATTTCCGGTGTAGCGGTGAAATGCATAGAGATCGGAAGGAACATCAGTGGCGAAGGCGGCACTCTGGCTTAAAACTGACGCTGAGGTACGAAAGCGTGGGTAGCAAACAGGATTAGATACCCTGGTAGTCCACGCCCTAAACGATGTCAACTAGCCGTTGGATCCATTTAAGGATTTAGTGGTGCAGCTAACGCATTAAGTTGACCGCCTGGGGAGTACGCACGCAAGTGTA | CM1 | 3 | *Thiotrichaceae* | GU369918_s | 99.2 | GU369918 | Uncultured gamma proteobacterium clone V1B07b16 | 99.20 | GU369918.1 | shallow hydrothermal Vents |
| TACGGAGGGTCCAAGCGTTATCCGGATTTATTGGGTTTAAAGGGTCCGTAGGCGGGGTTTTAAGTCAGTGGTGAAATCCTACAGCTCAACTGTAGAACTGCCATTGAAACTGGAACTCTTGAATGTGATTGAGGTAGGCGGAATATGTCATGTAGCGGTGAAATGCTTAGATATGCCATAGAACACCGATAGCGAAGGCAGCTTACCAAGTCATAATTGACGCTGATGGACGAAAGCGTGGGGAGCGAACAGGATTAGATACCCTGGTAGTCCACGCCGTAAACGATGATCACTAGCTATTGGCGATATACAGTCAGTGGCACAGCGAAAGTGTTAAGTGATCCACCTGGGGAGTACGATCGCAAGGTTG | CM1 | 2.6 | *Cryomorphaceae* | AY225660_s | 95.95 | AY225660 | Uncultured bacterium NZ-BA-8 | 98.11 | AB239762.1 | Brothers Caldera, south Kermadec Arc |
| TACGGAGGGTGCAAGCGTTACTCGGAATCACTGGGCGTAAAGCGCATGCAGGCGGTTTAATAAGTTAGAAGTGAAATCCTACAGCTTAACTGTAGAACTGCTTCTAAAACTGTTAGACTAGAGTCTGGGAGGGGAAGATGGAATTAGTAGTGTAGGGGTAAAATCCGTAGAGATTACTAGGAATACCAAAAGCGAAGGCGATCTTCTGGAACAGTACTGACGCTGAGATGCGAAAGCGTGGGGAGCAAACAGGATTAGATACCCTGGTAGTCCACGCAGTAAACGATGAATGTTAGTCGTCGGGGCACTAGTTGTTTCGGTGATGCAGTTAACACATTAAACATTCCGCCTGGGGAGTACGGTCGCAAGATTA | CM1 | 2.5 | *Sulfurovum* | AB278150_s | 96.51 | AB278150 | Uncultured bacterium clone G56 | 98.39 | JX568056.1 | marine hydrocarbon seep |
| TACGGAGGGGGTTAGCGTTGTTCGGAATTACTGGGCGTAAAGCGCACGTAGGCGGATTAGTCAGTCAGAGGTGAAATCCCAGGGCTCAACCCTGGAACTGCCTTTGATACTGCTAGTCTTGAGTTCGAGAGAGGTGAGTGGAATTCCGAGTGTAGAGGTGAAATTCGTAGATATTCGGAGGAACACCAGTGGCGAAGGCGGCTCACTGGCTCGATACTGACGCTGAGGTGCGAAAGCGTGGGGAGCAAACAGGATTAGATACCCTGGTAGTCCACGCCGTAAACGATGAATGCCAGACGTCGGGTAGCATGCTATTCGGTGTCACACCTAACGGATTAAGCATTCCGCCTGGGGAGTACGGTCGCAAGATTA | CM1 | 2.1 | *Actibacterium* | *Aliiruegeria sabulilitoris* | 98.92 | LOAS01000080 | Uncultured bacterium clone SAPTA-27 | 100.00 | MG251626.1 | tropical mangrove |
| TACGGAGGGTGCAAGCGTTATCCGGAATCATTGGGTTTAAAGGGTCCGTAGGCGGGTCATTAAGTCAGAGGTGAAATCCCACAGCTTAACTGTGGAACTGCCTTAGATACTGATGATCTTGAGTTTTAGTGAAGTAGATAGAATGTGTAGTGTAGCGGTGAAATGCATAGATATTACACAGAATACCGATTGCGAAGGCAGTCTACTAACTAACAACTGACGCTAATGGACGAAAGCGTGGGGAGCGAACAGGATTAGATACCCTGGTAGTCCACGCCGTAAACGATGGATACTAGTTGTTTGAGATTTATCTTGAGTGACTAAGCGAAAGTGATAAGTATCCCACCTGGGGAGTACGTTCGCAAGAATG | CM1 | 2 | *Maritimimonas* | *JQ579897_s* | 100 | JQ579897 | Uncultured Bacteroidetes bacterium clone FII-TR102 | 100.00 | JQ579897.1 | oil-polluted subtidal sediments |
| TACGGAGGGTGCAAGCGTTAATCGGAATTACTGGGCGTAAAGCGCGCGTAGGCGGCTTTGTAAGTCGGATGTGAAATCCCCGGGCTCAACCCGGGAACTGCATTCGATACTGCAGAACTAGAGTATGGTAGAGGGAAGTGGAATTCCGGGTGTAGCGGTGAAATGCGTAGATATCCGGAGGAACACCAGTGGCGAAGGCGACTTCCTGGGCCAATACTGACGCTGAGGTGCGAAAGCGTGGGGAGCAAACAGGATTAGATACCCTGGTAGTCCACGCCGTAAACGATGAGAACTAGATGTCGGGAGAATCTGTCTTTCGGTGTCGCAGCTAACGCGTTAAGTTCTCCGCCTGGGGAGTACGCCGGCAACGGTA | CM2 | 4.31 | *Woeseia* | *GU061281_s* | 97.86 | GU061281 | Uncultured bacterium clone s102 | 100.00 | AY171368.1 | marine sediment |
| TACGGGAGTGGCAAGCGTTATCCGGAATTATTGGGCGTAAAGCGTCCGCAGGCGGCCCTTCAAGTCTGCTGTTAAAAAGTGGAGCTTAACTCCATCATGGCAGTGGAAACTGTTGGGCTTGAGTGTGGTAGGGGCAGAGGGAATTCCCGGTGTAGCGGTGAAATGCGTAGATATCGGGAAGAACACCAGTGGCGAAGGCGCTCTGCTGGGCCATCACTGACGCTCATGGACGAAAGCCAGGGGAGCGAAAGGGATTAGATACCCCTGTAGTCCTGGCCGTAAACGATGAACACTAGGTGTCGGGGGAATCGACCCCCTCGGTGTCGTAGCCAACGCGTTAAGTGTTCCGCCTGGGGAGTACGCACGCAAGTGTG | CM2 | 3.5 | *Synechococcus_CC9902* | *CP006882_s* | 100 | CP006882 | Uncultured bacterium clone SCG_ch08 | 100.00 | MG736619.1 | ocean subsurface |
| TACGAAGGGACCTAGCGTAGTTCGGAATTACTGGGCTTAAAGAGTTCGTAGGTGGTTGAAAAAGTTGGTGGTGAAATCCCAGAGCTTAACTCTGGAACTGCCATCAAAACTTTTCAGCTAGAGTATGATAGAGGAAAGCAGAATTTCTAGTGTAGAGGTGAAATTCGTAGATATTAGAAAGAATACCAATTGCGAAGGCAGCTTTCTGGATCATTACTGACACTGAGGAACGAAAGCATGGGTAGCGAAGAGGATTAGATACCCTCGTAGTCCATGCCGTAAACGATGTGTGTTAGACGTTGGAAATTTATTTTCAGTGTCGCAGCGAAAGCGATAAACACACCGCCTGGGGAGTACGACCGCAAGGTTA | CM2 | 2.5 | *Clade_Ia* | *CP031125_s* | 100 | CP031125 | Candidatus Pelagibacter sp. FZCC0015 | 100.00 | CP031125.1 | seawater |
| TACGGAGGGTGCAAGCGTTGTCCGGATTTATTGGGTTTAAAGGGTGCGTAGGCGGCGTAACAAGTCAGTGGTGAAAGCCGGCAGCTCAACTGTCGAGGTGCCATTGAAACTATTATGCTTGAGTACAGACGAGGTAGGCGGAATTTATGATGTAGCGGTGAAATGCATAGATATCATAAAGAACACCGATAGCGAAGGCAGCTTACTAGGCTGTAACTGACGCTGAGGCACGAAAGCGTGGGGAGCGAACAGGATTAGATACCCTGGTAGTCCACGCTGTAAACGATGATGACTCGATGTTGGCGATAGACAGTCAGCGTCCTAGCGAAAGCGTTAAGTCATCCACCTGGGGAGTACGCTGGCAACAGTG | CM2 | 2.3 | *Cyclobacteriaceae* | *EU617868_s* | 98.92 | EU617868 | Uncultured Bacteroidetes bacterium clone T3-4 | 98.65 | KT880260.1 | microbial symbiont communities of the  sun sponge |
| TACGGAGGGTGCAAGCGTTACTCGGAATCACTGGGCGTAAAGCGCATGCAGGCGGTTTAATAAGTTAGAAGTGAAATCCTACAGCTTAACTGTAGAACTGCTTCTAAAACTGTTAGACTAGAGTCTGGGAGGGGAAGATGGAATTAGTAGTGTAGGGGTAAAATCCGTAGAGATTACTAGGAATACCAAAAGCGAAGGCGATCTTCTGGAACAGTACTGACGCTGAGATGCGAAAGCGTGGGGAGCAAACAGGATTAGATACCCTGGTAGTCCACGCAGTAAACGATGAATGTTAGTCGTCGGGGCACTAGTTGTTTCGGTGATGCAGTTAACACATTAAACATTCCGCCTGGGGAGTACGGTCGCAAGATTA | CM2 | 2.2 | *Sulfurovum* | *AB278150_s* | 96.51 | AB278150 | Uncultured bacterium clone G56 | 98.39 | JX568056.1 | seafloor hydrocarbon seep |
| TACAGAGGGTGCGAGCGTTAATCGGAATTACTGGGCGTAAAGCGCGCGTAGGCGGCTTGGTCAGTCGGATGTGAAAGCCCTGGGCTTAACCTGGGAATTGCATTCGATACTGCCAGGCTAGAATGTAGTAGAGGGAAGTGGAATTCCGGGTGTAGCGGTGAAATGCGTAGATATCCGGAGGAACATCAGTGGCGAAGGCGACTTCCTGGACTAACATTGACGCTGAGGTGCGAAAGCGTGGGGAGCAAACAGGATTAGATACCCTGGTAGTCCACGCCGTAAACGATGTCAACTAGATGTTGGGGGGTTTAACCCCTTAGTATCGCAGCTAACGCATTAAGTTGACCGCCTGGGGAGTACGGCCGCAAGGTTA | CM2 | 2.2 | *B2M28* | *HQ190975_s* | 98.93 | HQ190975 | Uncultured bacterium clone: MK0D_B22 | 99.73 | AB831353.1 | deep-sea methane-seep sediment |
| GACGAACCGTCCAAACGTTATTCGGTATCACTGGGCTTAAAGCGTGCGTAGGCGGCTTGGTAGGTGAGATGTGAAAGCCCACGGCTCAACCGTGGAATTGCGTTTCAAACCCCCAAGCTCGAGGAAGATAGGGGTGATGGGAACTTATGGTGGAGCGGTGAAATGCGTTGATATCATAGGGAACACCGGTGGCGAAAGCGCATCACTGGATCTTTTCTGACGCTGAGGCACGAAAGCTAGGGTAGCGAACGGGATTAGATACCCCGGTAGTCCTAGCCGTAAACGATGAACACTGGGTTGAGGGGACTTCCACATCCTCTCGGCCGTAGCGAAAGCGTTAAGTGTTCCGCCTGGGGAGTATGGTCGCAAGGCTG | CM2 | 2.2 | *Rubripirellula* | *Rubripirellula lacrimiformis* | 99.2 | MK559976 | Uncultured bacterium clone E133_A03 | 100.00 | KU578370.1 | ocean water |
| TACGTAGGTCCCGAACGTTGCGCGAATTTACTGGGCGTAAAGGGTCCGTAGGCGGTCTGGTAAGTGGAAGGTGAAATCCTGGGGCTCAACTCCAGAATTGCCTTCCAAACTGCTGGACTTGAGGCAGGGAGAGGTCGGCGGAATTCCCGGTGTAGCGGTGAAATGCGTAGATATCGGGAGGAACACCAGTGGCGAAGGCGGCCGACTGGAACTGTCCTGACGCTGAGGGACGAAAGCCAGGGGAGCGAACCGGATTAGATACCCGGGTAGTCCTGGCCGTAAACGATGGATGCTAGATGTGGGCAGGGAAACCTGTCCGTGTCGCAAGCTAACGCGTTAAGCATCCCGCCTGGGGAGTACGACCGCAAGGTTG | SF_G1 | 62.98% | *Hydrogenothermus* | *Hydrogenothermus marinus* | 99.2 | AJ292525 | Hydrogenothermus marinus strain VM1 | 98.93 | NR_114754.1 | Shallow water vents |
| CACGTAGGAGGCGAGCGTTACCCGGATTTACTGGGCGTAAAGCGCGCGCAGGCGGCTCGGTAAGTTGGGCGTGAAAGCTCCCGGCTCAACTGGGAGAGGACGTCCAAAACTGCCGGGCTAGAGGGCGGTAGAGGGAGGTGGAATTCCCGGTGTAGCGGTGAAATGCGTAGATATCGGGAGGAACACCAGTGGCGAAGGCGGCCTCCTGGACCGTCCCTGACGCTCAGGCGCGAAAGCCAGGGGAGCGAACGGGATTAGATACCCCGGTAGTCCTGGCCGTAAACGATGCGGACTAGGCGTTGGGCGGGTCAAACCGCTCAGTGCCGTAGCTAACGCGTTAAGTCCGCCGCCTGGGGACTACGGCCGCAAGGCTA | SF_G1 | 8.84% | *Anaerolineaceae* | *HQ727651_s* | 91.71 | HQ727651 | Uncultured bacterium clone J10_12x-E5_0239HSNP001F_P4 | 98.66 | JN838909.1 | shallow marine hydrothermal  Vent |
| TACGGAGGGTGCGAGCGTTACTCGGAATTACTGGGCGTAAAGGGCGCGTAGGCGGCTGGGCAAGTCTGGTGTGAAAGCCCGGGGCTCAACCTCGGAAGTGCACTGGATACTGTCTGGCTTGAGTACCGGAGAGGAGGGGGGAATTCCCGGTGTAGCGGTGAAATGCGTAGATATCGGGAGGAATACCGGTGGCGAAGGCGCCCCTCTGGACGGTAACTGACGCTGAGGCGCGAAAGCGTGGGGAGCAAACAGGATTAGATACCCTGGTAGTCCACGCTGTAAACGATGCCCACTAGGTGTGGTGGGGGTTAAGCCCTGCCGTGCCGTAGCTAACGCGTTAAGTGGGCCGCCTGGGGAGTACGGCCGCAAGGTTA | SF_G1 | 4.49% | *Thermodesulforhabdus* | *Thermodesulforhabdus norvegica* | 95.43 | U25627 | Thermodesulforhabdus sp. nov. M40/2 CIV-3.2 | 99.20 | AF170420.1 | geothermally heated sediments |
| TACGGAGGTGGCGAGCGTTGCCCGGAATCACTGGGCGTAAAGGGGGCGTAGGCGGCCAGGCAAGTCGGAGGTTAAAGCCCGGGGCTCAACCCCGGAAAGGCCTCCGATACTGCTTGGCTTGAGGGCCGGAGAGGCTGGCGGAATTCCCGGTGTAGGGGTGAAATCCGTAGATATCGGGAGGAACACCGGTGGGGAAGCCGGCCAGCTGGACGGTCCCTGACGCTGAGGCCCGAAAGCGTGGGGAGCAAACCGGATTAGATACCCGGGTAGTCCACGCCGTAAACGATGGGCGCTAGGTGTGGGGGGCTTTATCCCTCCGTGCCGTAGCTAACGCGTTAAGCGCCCCGCCTGGGGAGTACGGCCGCAAGGCTG | SF_G1 | 2.94% | *Thermosulfurimonas* | *Thermosulfurimonas dismutans* | 98.38 | LWLG01000001 | Thermosulfurimonas sp. strain F29 | 99.19 | MZ773229.1 | deep-sea hydrothermal vent |
| TACAGAGGGTGCAAGCGTTAATCGGAATTACTGGGCGTAAAGCGCGCGTAGGTGGTTTGATAAGTTGGATGTGAAATCCCCGGGCTTAACCTGGGTCGGTCATTCAAAACTGTCAGACTAGAGTATGGTAGAGGGTAGTGGAATTTCTAGTGTAGCGGTGAAATGCGTAGATATTAGAAGGAACACCAGTGGCGAAGGCGACTGCCTGGACTGATACTGACACTGAGGTGCGAAAGCGTGGGTAGCGAACAGGATTAGATACCCTGGTAGTCCACGCCGTAAACGATGACAACTAGACGTTGGGCTCCTTAGAGGGCTTAGTGTCGAAGCTAACGCGTTAAGTTGTCCGCCTGGGGAGTACGGTCGCAAGATTA | SF_G2 | 5.2 | *endosymbionts* | *Maorithyas hadalis gill thioautotrophic symbiont I/SB3-19* | 98.93 | AB188780 | Uncultured bacterium 5133BC_bac_p1B08 | 100.00 | KT280645.1 | Hydrate Ridge methane seep anaerobic Incubation |
| TACGGGAGTGGCAAGCGTTATCCGGAATTATTGGGCGTAAAGCGTCCGCAGGCGGCCCTTCAAGTCTGCTGTTAAAAAGTGGAGCTTAACTCCATCATGGCAGTGGAAACTGTTGGGCTTGAGTGTGGTAGGGGCAGAGGGAATTCCCGGTGTAGCGGTGAAATGCGTAGATATCGGGAAGAACACCAGTGGCGAAGGCGCTCTGCTGGGCCATCACTGACGCTCATGGACGAAAGCCAGGGGAGCGAAAGGGATTAGATACCCCTGTAGTCCTGGCCGTAAACGATGAACACTAGGTGTCGGGGGAATCGACCCCCTCGGTGTCGTAGCCAACGCGTTAAGTGTTCCGCCTGGGGAGTACGCACGCAAGTGTG | SF_G2 | 3.6 | *Synechococcus_CC9902* | *CP006882_s* | 100 | CP006882 | Uncultured bacterium clone 2015-11-17-SCG_ch08 | 100.00 | MG736619.1 | Sargasso Seawater |
| TACGGAGGGTGCGAGCGTTAATCGGAATTACTGGGCGTAAAGCGCGCGTAGGCGGTTATTTAAGTCGGATGTGAAATCCCCGGGCTCAACCTGGGAACTGCATTCGATACTGGGTAACTAGAGTCTGGTAGAGGGGGGTAGAATTCCTGGTGTAGCGGTGAAATGCGTAGATATCAGGAGGAATACCAGTGGCGAAGGCGGCCCCCTGGACCAAGACTGACGCTGAGGTGCGAAAGCGTGGGGAGCAAACAGGATTAGATACCCTGGTAGTCCACGCCGTAAACGATGTCAACTAGCCGTTGGGCCCATATAAGGGTTTAGTGGCGCAGCTAACGCAATAAGTTGACCGCCTGGGGAGTACGCCGGCAACGGTA | SF_G2 | 2.95 | *Gammaproteobacteria* | *FJ517003_s* | 93.58 | FJ517003 | Uncultured bacterium clone WP12Y7D | 95.45 | KX422147.1 | "hydrothermal sulfur vent micribial Mats |
| TACAGAGGGTGCAAGCGTTAATCGGAATTACTGGGCGTAAAGCGCGCGTAGGCGGTTTGTTAAGTCGGATGTGAAAGCCCCGGGCTCAACCTGGGAACTGCATTCGATACTGGCAGGCTAGAGTATGGTAGAGGGAAGTGGAATTCCGGGTGTAGCGGTGAAATGCGTAGATATCCGGAGGAACATCAGTGGCGAAGGCGGCTTCCTGGACCAATACTGACGCTGAGGTGCGAAAGCGTGGGGAGCAAACAGGATTAGATACCCTGGTAGTCCACGCCGTAAACGATGAGAACTAGACGTTGGGTTCATTTAAGGACTTAGTGTCGCAGCTAACGCGTGAAGTTCTCCGCCTGGGGAGTACGGCCGCAAGGTTA | SF_G2 | 2.92 | *Thiogranum* | *FM179879_s* | 98.66 | FM179879 | Uncultured bacterium clone BC4 | 99.73 | JX905991.1 | shallow hydrothermal vents |
| TACGGAGGGTGCAAGCGTTAATCGGAATTACTGGGCGTAAAGCGCGCGTAGGCGGCTTTGTAAGTCGGATGTGAAATCCCCGGGCTCAACCCGGGAACTGCATTCGATACTGCAGAACTAGAGTATGGTAGAGGGAAGTGGAATTCCGGGTGTAGCGGTGAAATGCGTAGATATCCGGAGGAACACCAGTGGCGAAGGCGACTTCCTGGGCCAATACTGACGCTGAGGTGCGAAAGCGTGGGGAGCAAACAGGATTAGATACCCTGGTAGTCCACGCCGTAAACGATGAGAACTAGATGTCGGGAGAATCTGTCTTTCGGTGTCGCAGCTAACGCGTTAAGTTCTCCGCCTGGGGAGTACGCCGGCAACGGTA | SF_G2 | 2.35 | *Woeseia* | *GU061281_s* | 97.86 | GU061281 | Uncultured bacterium clone s102 | 100.00 | AY171368.1 | marine sediment |
| TACGAAGGGACCTAGCGTAGTTCGGAATTACTGGGCTTAAAGAGTTCGTAGGTGGTTGAAAAAGTTGGTGGTGAAATCCCAGAGCTTAACTCTGGAACTGCCATCAAAACTTTTCAGCTAGAGTATGATAGAGGAAAGCAGAATTTCTAGTGTAGAGGTGAAATTCGTAGATATTAGAAAGAATACCAATTGCGAAGGCAGCTTTCTGGATCATTACTGACACTGAGGAACGAAAGCATGGGTAGCGAAGAGGATTAGATACCCTCGTAGTCCATGCCGTAAACGATGTGTGTTAGACGTTGGAAATTTATTTTCAGTGTCGCAGCGAAAGCGATAAACACACCGCCTGGGGAGTACGACCGCAAGGTTA | SF_G2 | 2.3 | *Clade_Ia* | *CP031125_s* | 100 | CP031125 | Candidatus Pelagibacter sp. FZCC0015 | 100.00 | CP031125.1 | seawater |
| TACGGAGGGTGCAAGCGTTAATCGGAATTACTGGGCGTAAAGCGCGCGTAGGCGGTTTGATAAGTCGGATGTGAAAGCCCTGGGCTCAACCTGGGAACTGCATTCGATACTGTCTGACTAGAGTATGGTAGAGGGAAGTGGAATTCCGGGTGTAGCGGTGAAATGCGTAGATATCCGGAGGAACATCAGTGGCGAAGGCGACTTCCTGGACCAATACTGACGCTGAGGTGCGAAAGCGTGGGGAGCAAACAGGATTAGATACCCTGGTAGTCCACGCCGTAAACGATGTCAACTAGCCGTTGGGGATATTAAAATCTTTAGTGGCGCAGCTAACGCGATAAGTTGACCGCCTGGGGAGTACGGTCGCAAGATTA | SF_G2 | 2.2 | *Thiohalophilus* | *EU652540_s* | 99.47 | EU652540 | Uncultured bacterium clone F1 NEREIS T270d | 100.00 | JF774447.1 | marine sediments microcosms |
| TACGGAGGGTCCAAGCGTTATCCGGATTTATTGGGTTTAAAGGGTCCGTAGGCGGGGTTTTAAGTCAGTGGTGAAATCCTACAGCTCAACTGTAGAACTGCCATTGAAACTGGAACTCTTGAATGTGATTGAGGTAGGCGGAATATGTCATGTAGCGGTGAAATGCTTAGATATGCCATAGAACACCGATAGCGAAGGCAGCTTACCAAGTCATAATTGACGCTGATGGACGAAAGCGTGGGGAGCGAACAGGATTAGATACCCTGGTAGTCCACGCCGTAAACGATGATCACTAGCTATTGGCGATATACAGTCAGTGGCACAGCGAAAGTGTTAAGTGATCCACCTGGGGAGTACGATCGCAAGGTTG | SF_G2 | 2.15 | *Cryomorphaceae* | *AY225660_s* | 95.95 | AY225660 | Uncultured bacterium sequence type NZ-BA-8 | 98.11 |  | cirral setae of Vulcanolepas osheai |
| TACGGAGGATTCGAGCGTTATCCGGATTTATTGGGTTTAAAGGGTCCGTAGGCGGGCGATTAAGTCAGTGGTGAAATCTCACAGCTCAACTGTGAAACTGCCATTGATACTGGTTGTCTTGAATTTAGTTGAGGTGGGCGGAATACGTTATGTAGCGGTGAAATGCATAGATATAACGTAGAACACCGATTGCGAAGGCAGCTCACTAAGCTAATATTGACGCTGATGGACGAAAGCGTGGGGAGCGAACAGGATTAGATACCCTGGTAGTCCACGCCGTAAACGATGATTACTCGTTGTGCGCGATACACAGTGCGCGACTGAGCGAAAGCATTAAGTAATCCACCTGGGGAGTACGTTGGCAACAATG | GB | 7.62 | *Marinifilum* | *Marinifilum fragile* | 100 | BAZX01000063 | Marinifilum fragile CECT 7942 strain JC2469 | 100.00 | NR_044597.2 | tidal flat Sediment |
| TACGTAGGGAGCAAGCGTTGTCCGGATTTACTGGGTGTAAAGGGCGCGCAGGCGGGTTGGTAAGTCAGAGGTGAAATCCTACAGCTTAACTGTAGAACTGCCTTTGATACTGCTGATCTTGAGTATGGAAGAGAGAGACGGAATTCCAGGTGTAGTGGTGAAATACGTAGATATCTGGAAGAACACCAGTTGCGAAGGCGGTCTCTTGGTCCAATACTGACGCTGAGGCGCGAAAGCGTGGGTAGCAAACAGGATTAGATACCCTGGTAGTCCACGCTGTAAACGATGAATACTAGGTGCTGGGTCTTTAGATTCAGTGTCGCAGCTAACGCATTAAGTATTCCACCTGGGGAGTACGATCGCAAGGTTG | GB | 5.87 | *PHOS-HE36* | *JQ580230_s* | 98.92 | JQ580230 | Uncultured bacterium clone APC-3439-J3F12 | 99.46 | KF616740.1 | Hydrate Ridge |
| TACGGAGGGGGCAAGCGTTATCCGGAATCACTGGGCGTAAAGAGCGCGTAGGCGGGTTAAAAAGTCGGGCGTGAAATTTATCGGCTTAACTGATAAATGTCGTCCGATACTTTTAATCTTGAGGATAGGAGAGGAGAGTAGAATTCCCGGTGTAGCGGTGAAATGCATTGATATCGGGAGGAATGCCAGTTGCGAAGGCGGCTCTCTGGAATATTCCTGACGCTGAGGCGCGAAAGCGTGGGTATCGAACCGGATTAGATACCCGGGTAGTCCACGCCGTAAACGATGGATGTTAGGTGTAGGGGGTTACTCCTGTGCCGTAGCTAACGCGTTAAACATCCCGCCTGGGGAGTACGGTCGCAAGGCTG | GB | 3.28 | *NA* | *ASOY_s* | 83.15 | ASOY01000050 | Uncultured bacterium clone 490CT10B38 | 84.55 | KX953010.1 | deep sea sediment |
| TACGGAGGGTGCAAGCGTTGTCCGGATTTATTGGGTTTAAAGGGTGCGTAGGCGGCGTAACAAGTCAGTGGTGAAAGCCGGCAGCTCAACTGTCGAGGTGCCATTGAAACTATTATGCTTGAGTACAGACGAGGTAGGCGGAATTTATGATGTAGCGGTGAAATGCATAGATATCATAAAGAACACCGATAGCGAAGGCAGCTTACTAGGCTGTAACTGACGCTGAGGCACGAAAGCGTGGGGAGCGAACAGGATTAGATACCCTGGTAGTCCACGCTGTAAACGATGATGACTCGATGTTGGCGATAGACAGTCAGCGTCCTAGCGAAAGCGTTAAGTCATCCACCTGGGGAGTACGCTGGCAACAGTG | GB | 2.53 | *Cyclobacteriaceae* | *EU617868_s* | 98.92 | EU617868 | Uncultured Bacteroidetes bacterium clone T3-4 | 98.65 | KT880260.1 | tidal creek sediment |
| CACGGGGGGGGCGAGCGTTATTCGGAATTACTGGGCGTAAAGGGCGCGTAGGCGGTCCGTTAAGTGTGAAGTGAAATGCCTGGGCTCAACCTGGGACGTGCTTTGCATACTGGTGGACTTGAGTCCAAGAGGGGGTGGTGGAATTCCTGGTGTAGGGGTGAAATCCGTAGATATCAGGAGGAACACCGTTGGCGAAGGCGGCCACCTGGATTGGTACTGACGCTGAGGCGCGAAAGCGTGGGGAGCGAACAGGATTAGATACCCTGGTAGTCCACGCCGTAAACGATGTTCACTTGGTGTTGGTGTGATTAACCACATCAATGCCGGAGCTAACGCATTAAGTGAACCGCCTGGGGAGTACGGTCGCAAGGCTG | GB | 2.53 | *Thermotomaculum* | *HQ916584_s* | 95.19 | HQ916584 | Uncultured bacterium clone FS396_454 | 100.00 | DQ909392.1 | marine hydrothermal vent fluids |
| TACGTAGGGGGCAAGCGTTGTCCGGATTTACTGGGTGTAAAGGGCGCGTAGGCGGGTTTGTAAGTCAGTGGTGAAATCCTACAGCTTAACTGTAGAACTGCCTTTGATACTGCAGACCTTGAGTACGGAAGAGAGAGGCGGAATTCCAGGTGTAGTGGTGAAATACGTAGATATCTGGAAGAACACCAGAGGCGAAGGCGGTCTCTTGGTCCGTTACTGACGCTGAGGCGCGAAAGCGTGGGGAGCAAACAGGATTAGATACCCTGGTAGTCCACGCCGTAAACGATGAATACTAGGTGTTGGGTTTTTAACTCAGTGCCGCAGCAAACGCATTAAGTATTCCACCTGGGGAGTACGATCGCAAGGTTG | GB | 2.17 | *IheB3-7* | *JQ579954_s* | 97.83 | JQ579954 | Uncultured bacterium clone D1-STK-23 | 98.37 | MH091110.1 | mangrove sediments |
| TACGGGGGGTGCAAGCGTTATTCGGATTTACTGGGCGTAAAGCGCGCGTAGGCGGCCGTTTAAGTCAGATGTGAAAGCCCGGGGCTCAACCCCGGAAGTGCATTTGATACTATTCGGCTTGAGTATGGGAGAGGGAAGTGGAATTCCTGGTGTAGAGGTGAAATTCGTAGATATCAGGAGGAACACCGGTGGCGAAGGCGACTTCCTGGACCAATACTGACGCTGAGGCGCGAAGGCGTGGGGAGCAAACAGGATTAGATACCCTGGTAGTCCACGCAGTAAACGGTGATCACTAGGTGTAGCGGGTATTGACCCCTGCTGTGCCGCAGCTAACGCATTAAGTGATCCGCCTGGGGAGTACGGCCGCAAGGTTA | GB | 2.1 | *Desulfosarcinaceae* | *FM242222_s* | 99.73 | FM242222 | Uncultured bacterium clone C158 | 100.00 | KJ817702.1 | soil |
| TACCCGCGGTCCAAGTCGCAGCCATTTTTATTGGGTCTAAAACATCCGTAGCTTGCTCTTTAAGTTCCTTGTGAAATCCTATATCTTAAATATAGGGCGTGCAGGGAATACTACTGAGCTAGAGACTGGAAGACGTAACGAGTACGTTCGAAGTAGCGGTTAAATGTGTTAATCTCGAGCGGACTAACAATAGCGAAGGCACGTTACGAGGACAGTTCTGACAGTAAGGGATGAAGGCTAGGGGCGCAAAACGGATTAGATACCCGTGTAGTCCTAGCAGTAAACACTGTACACTAAACATTAGTACCTCCTCGAGAGGTATTGGTGCTGAAGCGAAGGCGAAGAGTGTACTACCTGGGAAGTATAGTCGCAAGGCCG | GB | 2.07 | *SCGC_AAA011-D5* | *EU731474_s* | 94.18 | EU731474 | Uncultured archaeon O127906H12 | 94.97 | FN865760.1 | NA |
| TACGGAGGGTGCAAGCGTTATCCGGATTCATTGGGTTTAAAGGGTGCGCAGGCGGACTTTTAAGTCAGTGGTGAAATCCCGGGGCTCAACCCCGGAACTGCCATTGATACTGAAAGTCTTGAGTTTGGTTGAAGTAGGCGGAATGTAGCATGTAGCGGTGAAATGCTTAGATATGCTACAGAACACCGATCGCGAAGGCAGCTTACTAAACCAATACTGACGCTCAGGCACGAAAGCGTGGGGAGCGAACAGGATTAGATACCCTGGTAGTCCACGCCGTAAACTATGATCACTCGTTGTTGGCGATACACAGTCAGCGACCAAGCGAAAGCGATAAGTGATCCACCTGGGGAGTACGATCGCAAGGTTG | SF_EF | 5 | *Bacteroidetes_VC2.1_Bac22* | *AY197392_s* | 98.87 | AY197392 | Uncultured Cytophaga sp BJS72-056 | 100.00 | AB238986.1 | cold seep sediment |
| TACGGAGGATGCAAGCGTTATCCGGATTTATTGGGTTTAAAGGGTGCGCAGGCGGCCTTATAAGTCAGTGGTGAAATCTCTCGGCTCAACCGAGAAACTGCCATTGATACTGTAGGGCTAGAATACAGACGAGGTAGGCGGAATGTAGCATGTAGCGGTGAAATGCTTAGATATGCTACAGAACACCGATCGCGAAGGCAGCTTACCAGGCTGTTATTGACGCTAATGCACGAAAGCGTGGGGAGCGAACAGGATTAGATACCCTGGTAGTCCACGCCGTAAACGATGATCACTCGATGTTGGCGATAGACAGTCAGCGTCCAAGCGAAAGTATTAAGTGATCCACCTGGGGAGTACGATCGCAAGGTTG | SF_EF | 3.81 | *Lentimicrobiaceae* | *FJ497390_s* | 99.46 | FJ497390 | Uncultured Bacteroidetes bacterium clone VS_CL-139 | 99.46 | FJ497390.1 | Vailulu'u Seamount |
| TACGGGGGGAGCAAGCGTTGTCCGGAATTACTGGGCGTAAAGGGCGTGTAGGTGGGCTGATAAGTCAGATGTGAAAGCCCGCGGCTTAACCGCGGAACTGCATTTGAAACTGTCAGTCTTGAGTACGAGAGAGGGTAGTGGAATTCCCAGTGTAGCGGTGAAATGCGTAGATATTGGGAAGAACACCAGTAGCGAAGGCGGCTACCTGGCTCGCAACTGACGCTAATGCGCGAAAGCGTGGGGAGCAAACAGGATTAGATACCCTGGTAGTCCACGCTGTAAACGATGGGCACTAGGTGTCGGTTCCGCTTGCGGAATCGGTGCCGCAGCAAACGCATTAAGTGCCCCGCCTGGGGAGTACGATCGCAAGGTTG | SF_EF | 3.33 | *Caldithrix* | *AY280423_s* | 94.1 | AY280423 | Uncultured bacterium Acs2P47 | 98.66 | AB292968.1 | hydrothermal sulfide structure |
| TACGTAGGGGGCAAGCGTTGTCCGGATTTACTGGGTGTAAAGGGCGCGTAGGCGGGTTTGTAAGTCAGTGGTGAAATCCTACAGCTTAACTGTAGAACTGCCTTTGATACTGCAGACCTTGAGTACGGAAGAGAGAGGCGGAATTCCAGGTGTAGTGGTGAAATACGTAGATATCTGGAAGAACACCAGAGGCGAAGGCGGTCTCTTGGTCCGTTACTGACGCTGAGGCGCGAAAGCGTGGGGAGCAAACAGGATTAGATACCCTGGTAGTCCACGCCGTAAACGATGAATACTAGGTGTTGGGTTTTTAACTCAGTGCCGCAGCAAACGCATTAAGTATTCCACCTGGGGAGTACGATCGCAAGGTTG | SF_EF | 3.11 | *IheB3-7* | *JQ579954_s* | 97.83 | JQ579954 | Uncultured bacterium clone D1-STK-23 | 98.37 | MH091110.1 | mangrove sediments |
| TACGGAGGGTGCAAGCGTTAATCGGAATCACTGGGCGTAAAGCGTGCGTAGGCTGCGCTTCAAGTCAGACGTGAAAGCCCTCGGCTCAACCGAGGAATTGCGTTTGAAACTGGAGTGCTTGAGTCTCGGAGAGGTTGGCGGAATTCCTGGTGTAGGAGTGAAATCCGTAGATATCAGGAGGAACACCGGCGGCGAAGGCGGCCAACTGGACGAGTACTGACGCTGAGGTACGAAAGCGTGGGTAGCAAACAGGATTAGATACCCTGGTAGTCCACGCTGTAAACGATGGATATTAGGTGTCGGGGTTCACACTTCGGTGCCGCAGTTAACGCGTTAAATATCCCGCCTGGGGAGTACGGTCGCAAGGCTG | SF_EF | 2.72 | *Desulfovibrio* | *Pseudodesulfovibrio nedwellii* | 97.56 | LC752232 | Pseudodesulfovibrio sp. SYK | 97.30 | LC752232.1 | saline lake sediment |
| TACCGGCGGCTCGAGTGGTGGCCGCTATTACTGGGCTTAAAGCGTCCGTAGCTTGGTCGTTAAGTCTCTGGGGAAATCTTCCGGCTCAACCGGAAGGCGTCTCAGGGATACTGGCGGCCTAGGGATCGGGAGAGGTGAGAGGTACTCTGGGGGTAGGAGTGAAATCCTGTAATCCTCAGGGGACCACCTGTGGCGAAGGCGTCTCACCAGAACGACTCCGACAGTGAGGGACGAAAGCTGGGGGAGCAAACCGGATTAGATACCCGGGTAGTCCCAGCCGTAAACGATGCGCGTTAGGTGTATCGGTGACCACGAGTTACCGAGGTGCCGAAGGGAAACCGTGAAACGCGCCGCCTGGGAAGTACGGTCGCAAGGCTG | SF_EF | 2.71 | *Methanofollis* | *Methanofollis fontis* | 99.47 | MG437305 | Methanofollis fontis strain FWC-SCC2 | 99.47 | MG437305.1 | Methane marine sediment |
| TACGGAGGGTGCAAGCGTTACTCGGAATCACTGGGCGTAAAGCGCATGCAGGCGGTTTAATAAGTTAGAAGTGAAATCCTACAGCTTAACTGTAGAACTGCTTCTAAAACTGTTAGACTAGAGTCTGGGAGGGGAAGATGGAATTAGTAGTGTAGGGGTAAAATCCGTAGAGATTACTAGGAATACCAAAAGCGAAGGCGATCTTCTGGAACAGTACTGACGCTGAGATGCGAAAGCGTGGGGAGCAAACAGGATTAGATACCCTGGTAGTCCACGCAGTAAACGATGAATGTTAGTCGTCGGGGCACTAGTTGTTTCGGTGATGCAGTTAACACATTAAACATTCCGCCTGGGGAGTACGGTCGCAAGATTA | SF_EF | 2.5 | *Sulfurovum* | *AB278150_s* | 96.51 | AB278150 | Uncultured bacterium clone G56 | 98.39 | JX568056.1 | seafloor hydrocarbon seep |
| TACGGAGGATGCAAGCGTTATCCGGATTTATTGGGTTTAAAGGGTACGTAGGCGGAAAATTAAGTCAGTAGTGAAATCCTGCAGCTTAACTGTAGAACTGTTATTGATACTGGTTTTCTTGAATATAGTTGAGGTAGGCGGAATGTGTAATGTAGCGGTGAAATGCTTAGATATTACACAGAACACCGATTGCGAAGGCAGCTTACTAAGCTATGATTGACGCTGAGGTACGAAAGCGTGGGGAGCGAACAGGATTAGATACCCTGGTAGTCCACGCCGTAAACGATGATCACTCGTTGTTGGCAATACATCGTCAGCGACTGAGCGAAAGCATTAAGTGATCCACCTGGGGAGTACGCTCGCAAGAGTG | SF_EF | 2.1 | *Bacteroidetes_BD2-2* | *JF344511_s* | 99.19 | JF344511 | Uncultured Bacteroidales bacterium clone SD08_034 | 100.00 | KJ566252.1 | sediment |

**Supplementary table 2**. Envfit results against nMDS1, nMDS2- Jaccard weighted. One star (*); p-value less than 0.01, two stars (**) p-value is less than 0.001. Only variables with a p-value of less than 0.05 are marked in bold and considered statistically significant.

| **Variable** | **nMDS1** | **nMDS2** | **r^2^** | **Pr (>r)** |  |
| --- | --- | --- | --- | --- | --- |
| temp | 0.92386 | 0.38272 | 0.4129 | 0.1113 |  |
| **psu** | 0.40980 | 0.91218 | 0.8374 | 0.0018 | ** |
| do | 0.90785 | 0.41929 | 0.1342 | 0.5714 |  |
| orp | 0.54389 | 0.83916 | 0.3899 | 0.1254 |  |
| ph | -0.05997 | -0.99820 | 0.2043 | 0.3986 |  |
| **ms.cm** | 0.44242 | 0.89681 | 0.7311 | 0.0135 | * |
| mVph | 0.20780 | 0.97817 | 0.3928 | 0.1389 |  |
| **ppm.tds** | 0.44342 | 0.89631 | 0.7342 | 0.0134 | * |
| **Cl** | 0.42823 | 0.90367 | 0.7001 | 0.0159 | * |
| **Br** | 0.42841 | 0.90358 | 0.6867 | 0.0173 | * |
| SO4 | 0.62616 | 0.77969 | 0.3819 | 0.1260 |  |
| **Na** | 0.41695 | 0.90893 | 0.7149 | 0.0159 | * |
| NH3 | 0.30526 | 0.95227 | 0.3409 | 0.1890 |  |
| **K** | 0.65368 | 0.75677 | 0.7953 | 0.0314 | ** |
| **Mg** | 0.31056 | 0.95055 | 0.7771 | 0.1161 | ** |
| Ca | 0.91023 | 0.41411 | 0.1880 | 0.2997 |  |
| Mn | -0.54906 | 0.83578 | 0.3061 | 0.2246 |  |
| Fe | -0.60695 | 0.79474 | 0.3251 | 0.1971 |  |
| Zn | -0.36158 | -0.93234 | 0.1583 | 0.4973 |  |
| As | 0.76016 | 0.64974 | 0.3356 | 0.1860 |  |
| Rb | -0.06609 | 0.99781 | 0.2447 | 0.3202 |  |
| **Sr** | 0.62092 | 0.78388 | 0.6330 | 0.0201 | * |
| Mo | 0.74979 | 0.66168 | 0.1684 | 0.4646 |  |
| Cs | 0.90667 | 0.42183 | 0.3881 | 0.1342 |  |

**Supplementary table 3**. Envfit results against nMDS1, nMDS2- Jaccard unweighted. One star (*); p-value less than 0.05, two stars (**) p-value is less than 0.01. Only variables with a p-value of less than 0.05 are marked in bold and considered statistically significant.

| **Variable** | **nMDS1** | **nMDS2** | **r^2^** | **Pr (>r)** |  |
| --- | --- | --- | --- | --- | --- |
| temp | 0.84990 | 0.52695 | 0.4100 | 0.1257 |  |
| **psu** | 0.99650 | 0.08361 | 0.5575 | 0.0380 | * |
| do | 0.28411 | 0.95879 | 0.2883 | 0.2515 |  |
| orp | 0.69207 | 0.72183 | 0.3233 | 0.2077 |  |
| ph | -0.56141 | 0.82754 | 0.4068 | 0.1282 |  |
| ms.cm | 0.98627 | 0.16512 | 0.5031 | 0.0613 |  |
| mVph | 0.73503 | -0.67803 | 0.4937 | 0.0697 |  |
| ppm.tds | 0.98467 | 0.17444 | 0.5044 | 0.0612 |  |
| Cl | 0.99286 | 0.11930 | 0.4792 | 0.0739 |  |
| Br | 0.99393 | 0.10997 | 0.4719 | 0.0796 |  |
| SO4 | 0.92180 | 0.38765 | 0.3248 | 0.2007 |  |
| Na | 0.99463 | 0.10350 | 0.4827 | 0.0752 |  |
| K | 0.79606 | 0.60522 | 0.7080 | 0.1721 |  |
| Mg | 0.90212 | 0.43149 | 0.3885 | 0.1850 |  |
| Ca | 0.13836 | 0.99038 | 0.6906 | 0.9484 |  |
| Mn | 0.18578 | -0.98259 | 0.4590 | 0.0824 |  |
| Fe | 0.14126 | -0.98997 | 0.4838 | 0.0653 |  |
| Zn | -0.56923 | -0.82218 | 0.1064 | 0.6503 |  |
| As | 0.86097 | 0.50866 | 0.3213 | 0.2107 |  |
| Rb | 0.53325 | -0.84596 | 0.3308 | 0.2023 |  |
| **Sr** | 0.99571 | 0.09249 | 0.5824 | 0.0303 | * |
| Mo | 0.42101 | 0.90706 | 0.2559 | 0.3066 |  |
| Cs | 0.88616 | 0.46339 | 0.3857 | 0.1425 |  |
